# The impact of fear of attacks on pain‐related disability in cluster headache: Insights from the fear avoidance model

**DOI:** 10.1111/head.14823

**Published:** 2024-09-03

**Authors:** Janosch Fox, Charly Gaul, Mirjana Slijepcevic, Julia Ohse, Nicolina Peperkorn, Youssef Shiban

**Affiliations:** ^1^ Department of Psychology PFH Göttingen Göttingen Germany; ^2^ University Medical Centre Göttingen Göttingen Germany; ^3^ Medical Faculty University of Duisburg‐Essen Duisburg Germany; ^4^ Headache Center Frankfurt Frankfurt am Main Germany; ^5^ Benedictus Hospital Feldafing Feldafing Germany

**Keywords:** cluster headache, fear avoidance model, fear of attacks, pain‐related disability

## Abstract

**Objective:**

This study utilized the theoretical framework of the “fear avoidance model” (FAM) and investigated the role of fear of attack in pain‐related disability. To this end, a measurement specific to cluster headache (CH) was used to investigate whether fear of attacks, alongside attack frequency, is a significant predictor of pain‐related disability in CH.

**Background:**

Cluster headache substantially impacts daily functioning, yet empirical research exploring specific contributing factors is limited.

**Methods:**

A cross‐sectional online survey was undertaken in patients with CH, gathering sociodemographic, clinical data, and responses on the Cluster Headache Scale and the Depression, Anxiety and Stress Scale.

**Results:**

Analysis of data from 640 patients (chronic CH: 287/640 [44.8%]; female: 264/640 [41.3%]; male: 373/640 [58.3%]; gender diverse: three of 640 [0.5%]; age range: 18–86 years; mean [standard deviation] Cluster Headache Scales subscale disability score: 36.9 [9.8]; out of 869 respondents) revealed that both attack frequency and fear of attacks significantly predicted pain‐related disability (*p* < 0.001, percentage of variance explained: *R* ^2^ = 0.24). More variance was explained by fear of attacks (*R* ^2^ = 0.22) than by attack frequency (*R* ^2^ = 0.02). This relationship remained significant even when controlling for depression and anxiety, which were also identified as independent predictors of pain‐related disability (*p* < 0.001, *R* ^2^ = 0.44).

**Conclusion:**

This study emphasizes the relevance of psychological factors in CH‐related disability. Fear of attacks was found to be an independent predictor, while attack frequency was of minor relevance. Empirical investigation of the FAM in CH could improve the understanding of the mechanisms underlying disability and contribute to the development of CH‐specific interventions.

## Plain language Summary

In this study, we wanted to find out what makes cluster headache so disabling. We found that fear of cluster headache attacks plays an important role. Further research into psychological factors in cluster headache could help develop further interventions to reduce the burden on patients.

**Abbreviations**
CBT: cognitive behavioral therapy
CH: cluster headache
CHS: Cluster Headache Scale
DASS: Depression, Anxiety and Stress Scale
FAM: fear avoidance model
ICHD‐3: International Classification of Headache Disorders, third edition

## INTRODUCTION

Cluster headache (CH) is a primary headache disorder that affects about 0.1–2.0% of the population.^1^ CH is characterized by recurrent, very painful headache attacks that occur in clusters (episodic CH) or all the year without remissions of at least 3 months within a year (chronic CH).^2^ Cluster attacks often occur at night and have a seasonal pattern, occurring primarily in spring and fall.^3^

The impact of CH on daily life is profound, often affecting occupational functioning and social life. Available studies consistently indicate a reduced quality of life linked to the experience of CH.^4^, ^5^, ^6^ Moreover, patients with CH often experience psychological symptoms such as fear, aggression, sleep disturbances, and suicidal ideation, as well as psychiatric comorbidities such as anxiety disorders and depression.^6^, ^7^, ^8^, ^9^

Given the current limited understanding of the underlying causes of CH, the focus of pharmacological treatment is primarily on aborting acute pain attacks and on reducing both the frequency and severity of these attacks. To mitigate the negative impact of CH on mental health, elements of cognitive behavioral therapy (CBT) are used,^10^ but empirical studies on these therapy effects are lacking, and there are no psychological interventions that are specifically tailored to treat patients with CH.

In accordance with the bio‐psycho‐social model of illness,^11^ research on pain disorders has highlighted the contribution of psychological factors to the burden of disease. However, in CH the interaction of biological and psycho‐social factors has been insufficiently investigated and empirical studies focusing on the identification of psychological risk factors for functioning are limited.

The “fear avoidance model” (FAM)^12^ presents a theoretical framework that could substantially improve the understanding of how the burden of disease develops in CH. The FAM posits that dysfunctional cognitive responses to actual or anticipated pain, in particular pain‐related catastrophizing, can result in increased fear of pain. This fear, in turn, often leads to a range of maladaptive psychological and behavioral responses. These may include avoidance behaviors, hypervigilance towards pain, and dysfunctional resting. Over time, these maladaptive reactions contribute to increasing functional impairment. Applied to CH, the fear of cluster attacks would thus be a central element in the cascade postulated by the FAM leading to pain‐related disability.

While the FAM has been demonstrated with robust evidence in chronic musculoskeletal pain,^13^ its applicability in primary headache disorder is unclear. Research in patients with migraine reveals a strong link between pain‐related catastrophizing and more frequent and severe headache, increased psychological distress, and disability.^14^, ^15^, ^16^, ^17^, ^18^ In addition, negative affect and fear of attacks have been observed in patients experiencing migraine.^19^ Furthermore, anxiety was shown to be a risk factor for higher pain‐related disability.^20^

Although there are parallels with migraine research, the distinct pathophysiology and symptomatology of CH necessitate special investigations to assess the importance of the FAM for this condition. Empirical studies in CH are scarce and are often conducted using generic measures or scales developed specifically for migraine, which can lead to inaccuracies. Given that there is currently no cure for CH, there is a pressing need for interventions that reduce the negative impact on patients’ quality of life and functioning. Based on the principles derived from the FAM, this study was designed to investigate one potential mechanism underlying the development of disease burden in CH. To this end, the hypothesis that headache frequency and fear of attacks are predictors of pain‐related disability was tested using a CH‐specific measurement.

## METHODS

A systematic cross‐sectional survey was conducted from January to February 2023. Patients with a diagnosis of CH were asked to take part in this anonymous online survey. Diagnoses were re‐checked by asking the International Classification of Headache Disorders, third edition (ICHD‐3) criteria.^2^ The survey was hosted on the SoSci platform.^21^ This study was an a priori analysis of previously collected data that have not yet been published.

### Recruitment

Recruitment was carried out online via the websites of the Federal Association of Cluster Headache Self‐Help Groups (CGS e.V.) in Germany, the German Migraine and Headache Society (DMKG e.V.), and the Headache Center Frankfurt. In addition, patients with CH from the Headache Center Frankfurt and the Pain Clinic Kiel were recruited via email distribution lists as well as directly during clinic appointments.

### Ethics

The responsible Ethics Committee of the Bavarian Medical Association (BLÄK) was informed about the study in advance. As this study was based on the evaluation of anonymous questionnaires, no ethics approval was required (2022–1180). Participants gave their written informed consent prior to completing the questionnaire, and participation was voluntary. No financial compensation was offered for participating in the study.

### Power analysis

Based on the results of a meta‐analysis on the association between fear of pain and pain‐related disability,^22^ a sample size of at least *N* = 88 was calculated, assuming a mean effect size of *f* ^2^ = 0.15, a significance level of *α* = 0.05 and a test power of 0.90. With a conservatively estimated drop‐out rate of 50%, a sample size of *N* = 176 patients was aimed for. The power calculation was performed with G*Power version 3.1.9.7.

### Measures

The survey included the Cluster Headache Scale (CHS)^23^ to assess psychosocial factors and the German version of the Depression, Anxiety, and Stress Scale (DASS)^24^, ^25^ to assess psychological distress in patients. Moreover, headache‐related clinical characteristics (e.g., attack frequency and duration of illness) and sociodemographics were recorded. Participant gender was assessed as an item within the questionnaire; participants could indicate their gender identity as male, female, or diverse.

#### The DASS

The DASS^24^, ^25^ is a reliable and valid self‐report instrument for assessing psychological distress. The subscales showed high reliability (depression *α* = 0.88, anxiety *α* = 0.76, stress *α* = 0.86) and good discriminatory power (*rit* >0.30). The validity was confirmed by factor analysis and comparisons with established procedures.^25^ The three subscales measure the symptoms of depression, anxiety, and stress with seven items each, resulting in a total of 21 items. Respondents rate each item on a 4‐point Likert scale (0 = Did not apply to me at all, 1 = Applied to me to some degree, or some of the time; 2 = Applied to me to a considerable degree, or a good part of the time; 3 = Applied to me very much, or most of the time) for the period of the last week.^24^, ^25^ According to the authors, the exclusion of somatic items makes the questionnaire suitable for the assessment of psychological stress in the research and treatment of patients with pain.^26^

#### The CHS

The CHS^23^ is a recently developed, reliable, and valid self‐report instrument for assessing disease‐specific psychosocial factors in persons with CH. It enables the calculation of both a total score and individual scores for each of the eight subscales, namely medical care, side‐effects of medication, fear of attacks, disability, (auto)aggression, coping, physical activity, and financial burden. Respondents rate their level of agreement for each of the 36 items on a 5‐point Likert scale (1 = strongly disagree; 2 = disagree; 3 = neither agree nor disagree; 4 = agree; 5 = strongly agree). An overview of the items of the subscales “Disability” and “Fear of Attacks” can be found in the Appendix. The scales showed acceptable to excellent internal consistencies (*α* = 0.76–0.93). Convergent validity is supported by significant correlations with related instruments.

### Statistical analysis

Statistical analyses were performed using the Statistical Package for the Social Sciences (SPSS, version 28; IBM Corp., Armonk, NY, USA). Measures of central tendency (mean or median), measures of variability (standard deviation or interquartile range) and frequency distributions (absolute [*N* or *n*] and relative frequency [%]) were used to summarize the characteristics of the study population and outcomes. The effect sizes of correlations are reported according to the conventions set by Cohen^27^; a correlation coefficient of 0.1 is considered a weak correlation, 0.3 a moderate correlation, and ≥0.5 a strong correlation. A *p* < 0.05 was considered statistically significant for all analyses. To counteract the accumulation of alpha errors, which could lead to false‐positive results, the Bonferroni correction was applied to adjust the significance level for multiple testing. To ensure the integrity and reliability of the analysis, participants with incomplete data sets were excluded.

To test the directional hypothesis, a hierarchical linear regression was conducted. In regression Model 1, the relationship between fear of attacks and disability was examined. To control for potential confounders, attack frequency (Model 2) as well as depression and anxiety (Model 3) were included as covariates. Headache frequency was defined as the reported average number of CH attacks/week. The CHS subscales “Fear of attacks” and “Disability” were used to measure fear of attacks and pain‐related disability (see Appendix for items). Data analysis found no outliers, as evidenced by a Cook's distance of <1^28^ and a leverage of <0.02.^29^ To account for heteroscedasticity, robust standard errors were calculated using the HC4 method.^30^ To review our data analysis, we consulted with the Department of Medical Statistics at the University Medical Center Göttingen.

## RESULTS

### Study population

**FIGURE 1:**
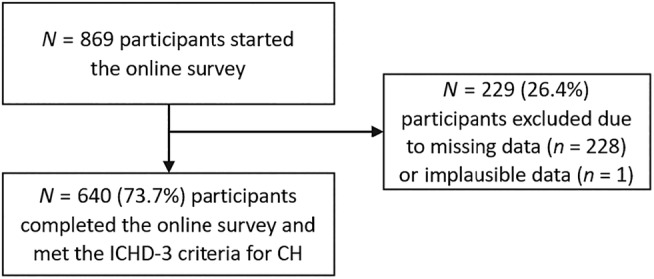
Flow chart of attrition. CH, cluster headache; ICHD‐3, International Classification of Headache Disorders, third edition.

Complete data sets of 640 of 869 participants could be included (Figure 1). Missing data due to a lack of completion in the survey was observed in 228/869 cases. One case had to be excluded due to implausible data (reported current age was below the indicated age at the time of diagnosis). All participants who completed the online survey met the ICHD‐3^2^ criteria for CH. A preliminary dropout analysis using incomplete data sets from 165/228 (72.4%) patients indicated no significant differences in age, gender, attack frequency, and course of disease between the included participants and those with missing data.

### Sociodemographic and clinical characteristics

Participants’ sociodemographic and clinical characteristics are summarized in Table 1. The mean (standard deviation, range) age of the participants was 46.8 (12.1, 18–86) years, with the majority identifying as male (58% [*n* = 373]).

**TABLE 1:** Sociodemographic and clinical characteristics of the study population (*N* = 640).

Characteristic	Value
Age, years, mean (SD)	46.8 (12.1)
Sex, *n* (%)	
Male	373 (58.3)
Female	264 (41.3)
Diverse	3 (0.5)
Education, *n* (%)	
Without graduation	5 (0.8)
Graduated 9th grade	118 (18.4)
Graduated 10th grade	192 (30)
High school diploma	168 (26.3)
Academic degree	157 (24.5)
Course of disease, *n* (%)	
Episodic CH	353 (55.2)
Chronic CH	287 (44.8)
CH history,^a^ years since diagnosis, mean (SD)	10.8 (8.9)
Attack frequency, median (IQR)	12.0 (7.0–21.0)
CHS sum score, mean (SD) [cluster attacks/week]	112.3 (21.3) [36–180]
CHS fear of attacks score, mean (SD) [cluster attacks/week]	15.8 (3.7) [4–20]
CHS disability score, mean (SD) [cluster attacks/week]	36.9 (9.8) [11–55]
DASS depression score, mean (SD) [cluster attacks/week]	8.5 (6.2) [0–21]
DASS anxiety score, mean (SD) [cluster attacks/week]	7.0 (5.1) [0–21]
DASS stress score, mean (SD) [cluster attacks/week]	10.6 (6.1) [0–21]

Abbreviations: CH, cluster headache; CHS, Cluster Headache Scale; DASS, Depression, Anxiety and Stress Scale; IQR, interquartile range; *n*, number valid cases.

^a^ *n* = 638.

Slightly more than half of the participants met the ICHD‐3 criteria for episodic CH (55% [*n* = 353]). The reported average frequency of cluster attacks per week was a median (interquartile range) of 12.0 (7.0–21.0) attacks/week. The total number of cluster attacks per week ranged from 1 to 95, whereby >56 attacks/week was reported in only seven cases.

In terms of psychosocial burden due to CH, an above‐average burden (CHS total score ≥133) was found in 17.7% of participants. Compared to the norm sample of the CHS, 16.4% (*n* = 105) reported above‐average disability (score >46) and 18% (*n* = 115) reported above‐average fear of attacks (score >19).

Regarding psychological distress, 42.1% of participants scored ≥10 on the DASS depression subscale, 54.8% of participants scored ≥6 on the DASS anxiety subscale, and 54.0% of participants scored ≥10 on the DASS stress subscale, indicating screening results above the norm.

### Zero‐order correlations between headache and psychological outcome parameters

Zero‐order correlations between headache outcome parameters (attack frequency, duration of illness) and psychological outcome parameters (fear of attacks, depression, anxiety, stress, psychosocial burden, disability) are shown in Table 2. Concerning the psychological outcomes, there were low correlations with attack frequency and medium correlations with fear of attacks. These results indicate the patients with higher scores on fear of attacks also indicated higher scores on pain‐related disability, depression, anxiety, stress and overall CH‐specific psychosocial distress. Duration of illness was not significantly correlated with attack frequency or attack anxiety.

**TABLE 2:** Zero‐order correlations between cluster headache variables and psychological outcome variables (*N* = 640).

Outcome parameter	Attack frequency	CHS subscale fear of attacks
Attack frequency		**0.09** *
Duration of illness in years	0.07	−0.09
DASS depression subscale	**0.13** **	**0.44** ***
DASS anxiety subscale	**0.11** **	**0.42** ***
DASS stress subscale	**0.09** *	**0.40** ***
CHS sum score	**0.20** ***	**0.47** ***
CHS fear of attacks subscale	**0.09** *	
CHS disability subscale	**0.20** ***	**0.47** ***

*Note*: Spearman's rho is given. Significant correlations are marked in bold.

Abbreviations: CHS, Cluster Headache Scale; DASS, Depression, Anxiety and Stress Scale.

* *p* < 0.05;

** *p* < 0.01;

*** *p* < 0.001.

### Frequency of fear of attacks

To assess the frequency of fear of attacks in the study population, three items of the CHS subscale “fear of attack” (items 9, 10, 11) were evaluated in dichotomous form: The responses “strongly agree” and “agree” were combined to “agree,” “strongly disagree”, “disagree,” and “neither agree or disagree” were combined to “disagree”. In all, 80.3% of the participants stated that they were afraid of a CH attack, 70.2% stated they were worried that an attack could occur, and 53.2% reported panic when thinking about the next attack.

### Prediction of pain‐related disability by headache frequency and fear of attacks

Two multiple linear regression analyses were conducted to examine the relationship between fear of attacks and frequency of attacks with pain‐related disability. In addition, a third multiple linear regression analysis was performed to evaluate the influence of depression and anxiety on pain‐related disability. The results for all three models tested are shown in Table 3.

**Model 1** explained 22% of variance in pain‐related disability (*R* ^2^ = 0.22), indicative of a medium to high goodness‐of‐fit according to Cohen.^27^ Fear of attacks was shown to be a significant predictor of pain‐related disability, *F*(1, 638) = 178.07, *p* < 0.001.
**Model 2** explained 24% of variance in pain‐related disability (*R* ^2^ = 0.24, adjusted *R* ^2^ = 0.24, change in *R* ^2^ = 0.03), indicative of a medium to high goodness‐of‐fit according to Cohen.^27^ Fear of attacks and the frequency of attacks were found to be independent predictors of pain‐related disability, *F*(2, 637) = 102.9, *p* < 0.001.
**Model 3** included depression and anxiety as additional predictors. This model showed an improvement by explaining 44% of the variance (*R* ^2^ = 0.44, adjusted *R* ^2^ = 0.44, change in *R* ^2^ = 0.20) in pain‐related disability, which is indicative of a high goodness‐of‐fit, according to Cohen.^27^

**TABLE 3:** Results of the multiple linear regression analyses for the variance analysis of pain‐related disability (*N* = 640).

Model	*R*	*R* ^2^	*R* ^2^_adj_	*p*	*SE*_*B*_	*B*	*β*
**Model 1**	0.47	0.22	0.22	<0.001			
Intercept				<0.001	1.69	17.39	
Fear of attacks				<0.001	0.10	1.24	0.47
**Model 2**	0.50	0.24	0.24	<0.001			
Intercept				<0.001	1.70	16.04	
Fear of attacks				<0.001	0.10	1.20	0.45
Attack frequency				<0.001	0.03	0.12	0.16
**Model 3**	0.66	0.44	0.44	<0.001			
Intercept				<0.001	1.48	18.96	
Fear of attacks				<0.001	0.10	0.59	0.22
Attack frequency				<0.001	0.02	0.09	0.12
Depression				<0.001	0.08	0.53	0.33
Anxiety				<0.001	0.09	0.40	0.21

Abbreviation: SE_B_, robust standard error (HC4).

Model 3 presented a mean disability score of 18.96 in the absence of fear of attacks, depression and anxiety, and cluster attacks. For each point extra on the fear of attacks scale, the mean disability increased by 0.59. A 1‐point increase on the depression scale contributed to a mean disability increase of 0.53, while a 1‐point gain on the anxiety scale increased mean disability by 0.40. Furthermore, the disability score increased by 0.09 with each reported attack.

All added variables were found to be independent predictors of disability, *F*(4, 635) = 124.83, *p* < 0.001, which could be confirmed by subsequent univariate analyses (fear of attack: *F*[1, 638] = 178.07, *p* < 0.001; frequency of attacks: *F*[1, 638] = 27.04, *p* < 0.001; depression: *F*[1, 638] = 352.62, *p* < 0.001; anxiety: *F*[1, 638] = 285.36, *p* < 0.001). The results of the uni‐ and multivariate analyses remained significant even when the Bonferroni correction for multiple testing was applied (corrected *p* value for all analyses: *p* = 0.003).

**FIGURE 2:**
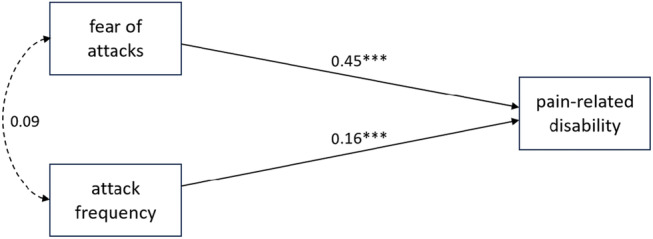
Sketch of the relationship between fear of attacks, frequency of attacks and pain‐related disability. Standardized regression coefficients between independent and dependent variables and zero‐order correlation between predictors are illustrated. ***p < 0.001. *N* = 640.

**FIGURE 3:**
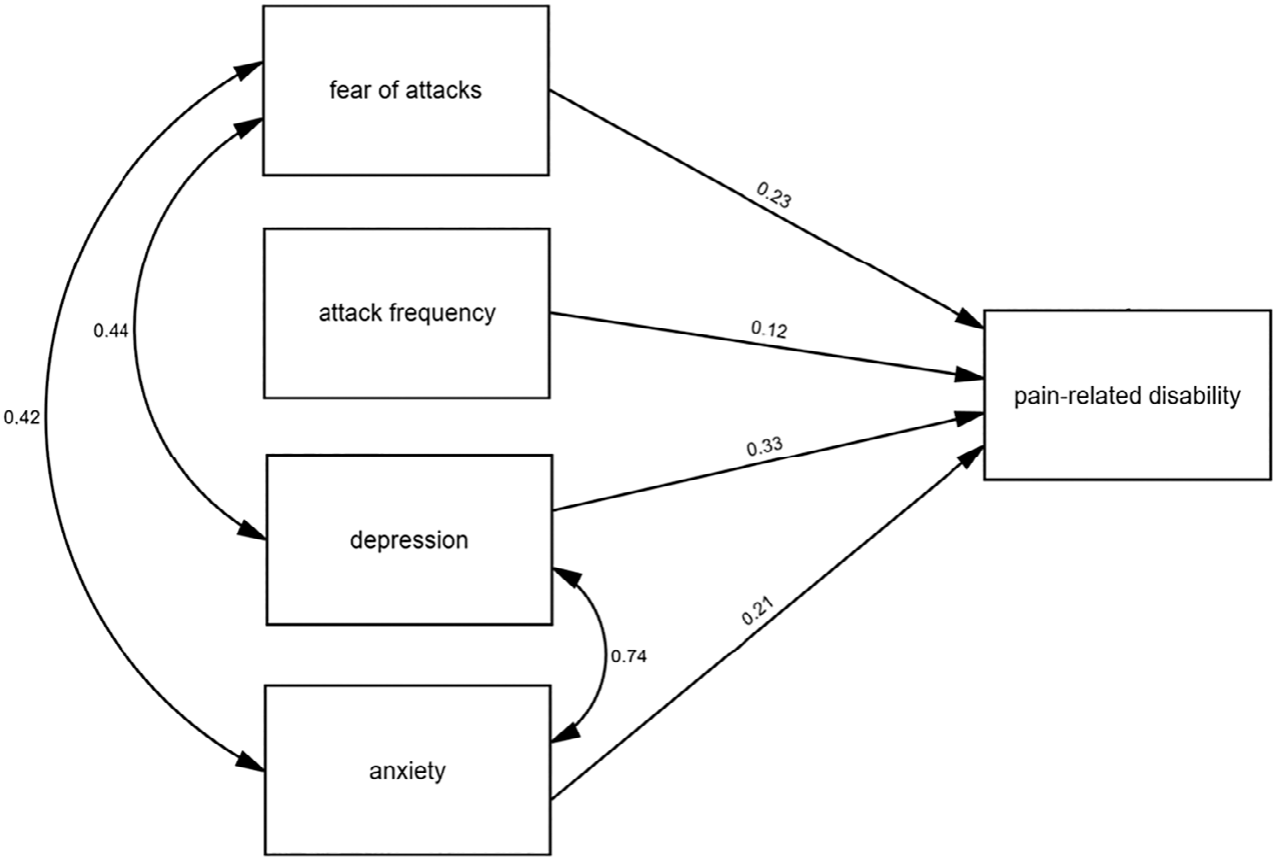
Exploratory path analysis of the relationship between fear of attacks, attack frequency, depression, anxiety, and pain‐related disability. Single‐headed arrows represent standardized regression weights, double‐headed arrows show correlations. Good model fit *χ* ^2^ = 11.02, *df* = 3, *p* = 0.012; Comparative Fit Index = 0.99; standardized Root Mean Square Residual = 0.05). All coefficients are significant (*p* < 0.001). Bootstrapping with 2000 samples was performed. All variables were observed. Residual variances are not shown for reasons of clarity. *N* = 640.

Figure 2 shows the relationship between the dependent and independent variables from Model 2. Fear of attack and attack frequency remained independent predictors of disability, even when depression and anxiety were included as control variables in Model 3. Figure 3 shows the results of an exploratory path analysis with good model fit (*χ* ^2^ = 11.02, *df* = 3, *p* = 0.012; Comparative Fit Index = 0.99; standardized Root Mean Square Residual = 0.05). Single‐headed arrows represent standardized regression weights, double‐headed arrows show correlations. All independent variables were significantly associated with disability (*p* < 0.001). Significant correlations among the predictors were observed between fear of attacks and depression (*p* < 0.001), fear of attacks and anxiety (*p* < 0.001), as well as between depression and anxiety (*p* < 0.001). No significant correlations were found between the attack frequency and other predictors in this path analysis.

## DISCUSSION

Based on the theoretical framework of the FAM, this study investigated the relationship between fear of attacks, attack frequency, and pain‐related disability in CH, utilizing a CH‐specific measurement instrument. Both fear and frequency of attacks were found to predict pain‐related disability, which is consistent with the assumptions of the FAM. Remarkably, fear of attacks contributed more to the variance explanation than frequency of attacks and remained an independent predictor even when controlling for depression and anxiety. Our findings lend support to the theoretical framework of the FAM and provide further evidence that psychological factors play a crucial role in CH‐related disability.

First, we found a significant association between fear of attacks and disability, which is consistent with existing findings on pain disorders in general^22^ and other primary headache disorders.^31^ In CH, an analysis as part of the EUROLIGHT project^32^ found a relationship between fear or worry about the next cluster attack and a reduced quality of life.^33^ While a relationship between attack frequency and disability could be found, this association was found to be relatively weak. Therefore, the number of attacks contributed significantly less to the explanation of disability than fear of attacks.

Second, our study showed that fear of attacks remained a positive predictor of disability, even when controlling for depression and anxiety. Depression and anxiety were found to be independent predictors in our study, which is in line with the findings of Black et al. (2015)^31^ in migraine.

Taken together, these results support the assumption of the FAM that functional impairment cannot be explained by attack frequency alone in terms of a simple dose–response relationship. Instead, it appears that other factors have a substantial influence on this relationship in terms of CH. In our study, we were able to identify three such psychological factors (fear of attacks, depression, and anxiety) that had a significant impact on pain‐related disability. Although these three factors were associated with each other, they were also found to be independent predictors of disability. This supports the notion that fear of attack is an independent construct that appears to coexist with negative affect, as indicated by Rogers et al.^34^

Moreover, low zero‐order correlations between attack frequency and psychological outcomes (fear of attacks, depression, and anxiety) could not be confirmed by an exploratory path analysis conducted in this study. Interaction effects and/or other factors (e.g., dysfunctional cognitions as postulated by the FAM) may, therefore, play a role in the increase in pain‐related disability.

Using a CH‐specific measurement instrument, we observed a high prevalence of fear of attacks within our sample. Four‐fifths of the participants stated being afraid of a CH attack, about two‐thirds were worried that an attack could occur, and one‐half reported panic when thinking about the next attack.

As anxiety can be treated by psychological interventions,^35^ fear of attacks could also be modified through such interventions. It appears conceivable that, in addition to standard CH treatment, specific interventions targeting fear of attacks could improve the quality of life of patients with greater disability.

In the context of migraine treatment, Martin et al.^36^, ^37^, ^38^ have already developed a promising psychotherapeutic approach that focuses on the modification of dysfunctional trigger avoidance behavior due to fear of attacks. This is a case representative of an adaptation of the FAM to a specific primary headache disorder. However, the effectiveness of integrating this approach into a CBT program has not yet been conclusively proven.^39^ Furthermore, trigger management appears to play a more limited role in CH than in migraine, for which specific CBT programs have already been developed and evaluated.^40^

According to the FAM, dysfunctional cognitions, such as “It's terrible and I think it's never going to get any better,” “There's nothing I can do to reduce the intensity of the pain,” or “I wonder whether something serious may happen” (example items from the Pain Catastrophizing Scale^41^), contribute notably to the progression towards disability. Recent research in migraine indicates mindfulness‐based therapies, as well as acceptance and commitment therapy, can lead to improvement in disability even if headache frequency remains unchanged.^42^ This suggests that these more modern CBT approaches, which focus on a functional level without targeting the pain itself, may also be effective in CH. Further research on pain catastrophizing and its impact on other aspects of the FAM could, therefore, improve our understanding of the mechanisms underlying disability and contribute to the development of CH‐specific interventions.

This study's strength lies in its use of a measurement instrument specifically designed for CH, enhancing the accuracy of the findings. The theory‐driven methodology and substantial sample size support the results’ validity. To ensure the integrity and reliability of the analysis, participants with incomplete data sets were excluded. Despite this exclusion, our final sample size remained robust, and preliminary dropout analysis indicated no significant differences in the sociodemographic and clinical characteristics between included participants and those with missing data, thus minimizing concerns about selection bias.

Nonetheless, the study's cross‐sectional design poses a notable limitation. Furthermore, patient‐reported data were used to assess outcomes and clinical characteristics, which might lead to bias if obtained without medical guidance.^43^ Additionally, the actual participation rate could not be determined, as the diversified recruitment strategies made it impossible to track how many participants in total were invited to take part in the survey. Within our sample, 41.4% of participants identified as female, which is a relatively high percentage compared to the male‐to‐female ratio reported in previous literature.^44^ It is not clear whether this can be attributed to a selection effect, as recent findings^45^ suggest a shift in epidemiology with more females experiencing CH.

To evaluate the impact of fear of attacks and pain catastrophizing on other components of the FAM and psychological outcomes, future research could explore manipulating these two critical FAM elements in longitudinal studies or randomized controlled trials.

## CONCLUSION

Fear of attacks appears to have a relevant influence on pain‐related disability in CH, which is in line with the FAM. A better understanding of the complex interplay of biological and psychosocial factors in CH may lead to the development of tailored interventions that reduce the adverse consequences of CH on functioning and quality of life. The FAM can serve as a theoretical framework.

## AUTHOR CONTRIBUTIONS

**Janosch Fox:** Conceptualization; data curation; formal analysis; methodology; project administration; visualization; writing – original draft; writing – review and editing. **Charly Gaul:** Supervision; writing – review and editing. **Mirjana Slijepcevic:** Investigation; resources. **Julia Ohse:** Writing – review and editing. **Nicolina Peperkorn:** Writing – review and editing. **Youssef Shiban:** Supervision; validation; visualization; writing – review and editing.

## FUNDING INFORMATION

We acknowledge support by the Open Access Publication Fund of the University of Duisburg‐Essen.

## CONFLICT OF INTEREST STATEMENT

**Charly Gaul** has received honoraria for consulting and lectures within the past 3 years from Abbvie, Lilly, Novartis Pharma, Hormosan Pharma, Grünenthal, Sanofi‐Aventis, Lundbeck, Perfood, Vectura Fertin Pharma, betapharm, Reckitt, Cordate and TEVA. His research is supported by a grant from the German Research Foundation (DFG). He does not hold any stocks in pharmaceutical companies. **Janosch Fox, Mirjana Slijepcevic, Julia Ohse, Nicolina Peperkorn,** and **Youssef Shiban** declare no conflicts of interest.

## APPENDIX A

Items of the CHS subscales “Disability” and “Fear of attacks”.

**Table:**

CHS Subscale “Disability”	CHS Subscale “Fear of attacks”
The CH disorder is determining my life. I feel limited in daily life. The CH disorder determines my daily routine. I am lonely because of my CH disorder. I withdraw socially because of my CH disorder. I suffer from the limitations caused by my CH disorder. I am limited in my scope of action due to my CH disorder. My vacation planning is affected by the disorder. My family life is impaired. My social contacts have become less due to my disorder. My social environment suffers from my illness.	I am afraid that the disorder could get worse. I am worried that the disorder could get worse. I am afraid of an CH attack. I am worried that an attack might occur. I panic when I think about the next attack. I am worried about the next attack.

Abbreviations: CH, cluster headache; CHS, Cluster Headache Scale.
